# Development and Validation of the Bicultural Youth Acculturation Questionnaire

**DOI:** 10.1371/journal.pone.0161048

**Published:** 2016-08-24

**Authors:** Atif Kukaswadia, Ian Janssen, William Pickett, Jasmine Bajwa, Katholiki Georgiades, Richard N. Lalonde, Elizabeth C. Quon, Saba Safdar, Ian Pike

**Affiliations:** 1 Department of Public Health Sciences, Queen’s University, Kingston, Ontario, Canada; 2 School of Kinesiology and Health Studies, Queen’s University, Kingston, Ontario, Canada; 3 Clinical Research Centre, Kingston General Hospital, Kingston, Ontario, Canada; 4 Department of Educational Psychology, University of Alberta, Edmonton, Alberta, Canada; 5 Offord Centre for Child Studies, McMaster University, Hamilton, Ontario, Canada; 6 Faculty of Health & Glendon College, York University, Toronto, Ontario, Canada; 7 IWK Health Centre, Halifax, Nova Scotia, Canada; 8 Department of Psychology, University of Guelph, Guelph, Ontario, Canada; 9 Department of Pediatrics, Faculty of Medicine, University of British Columbia and BC Injury Research and Prevention Unit, Child and Family Research Institute, BC Children’s Hospital, Vancouver, Canada; Philipps-Universitat Marburg, GERMANY

## Abstract

**Objectives:**

Acculturation is a multidimensional process involving changes in behaviour and beliefs. Questionnaires developed to measure acculturation are typically designed for specific ethnic populations and adult experiences. This study developed a questionnaire that measures acculturation among ethnically diverse populations of youth that can be included as a module in population surveys.

**Methods:**

Questionnaires measuring acculturation in youth were identified in the literature. The importance of items from the existing questionnaires was determined using a Delphi process and this informed the development of our questionnaire. The questionnaire was then pilot tested using a sample of 248 Canadians aged 18–25 via an online system. Participants identified as East and South East Asian (27.8%), South Asian (17.7%) and Black (13.7%). The majority were 1^st^ (33.5%) or 2^nd^ generation immigrants (52.0%). After redundant items were eliminated, exploratory factor analysis grouped items into domains, and, for each domain, internal consistency, and convergent validity with immigrant generation then age at immigration estimated. A subset of participants re-completed the questionnaire for reliability estimation.

**Results:**

The literature review yielded 117 articles that used 13 questionnaires with a total of 440 questions. The Delphi process reduced these to 32 questions. Pilot testing occurred in 248 Canadians aged 18–25. Following item reduction, 16 questions in three domains remained: dominant culture, heritage language, and heritage culture. All had good internal consistency (Cronbach’s alphas > .75). The mean dominant domain score increased with immigrant generation (1^st^ generation: 3.69 (95% CI: 3.49–3.89), 2^nd^: 4.13 (4.00–4.26), 3^rd^: 4.40 (4.19–4.61)), and mean heritage language score was higher among those who immigrated after age 12 than before (p = .0001), indicative of convergent validity.

**Conclusions:**

This Bicultural Youth Acculturation Questionnaire has demonstrated validity. It can be incorporated into population health surveys to elucidate the impact of acculturation on health outcomes among bicultural youth.

## Background

Acculturation occurs when two cultures meet, such as when an individual immigrates to a new country.[1] This is a multidimensional process of behavioural and psychological change that may give rise to changes in health and health-related behaviours.[2–6] However, measurement of acculturation in a quantitative manner is difficult. The bidimensional theory of acculturation hypothesizes that this process occurs over two dimensions, with individuals finding a balance between retention of heritage culture and, separately, adoption of dominant culture norms and values along a series of domains, with the dominant culture defined as those who hold the language, social, and cultural norms of the “receiving society.”[1,7,8] Because of this complexity, proxy indicators for acculturation such as “immigrant generation” or “age at immigration” are often used as they are easier to obtain.[9,10] However, they have limited content validity and do not capture the full spectrum over which acculturation can occur.[11,12]

A number of questionnaires have been developed to capture various dimensions and domains of acculturation among youth. However, approximately 61% were developed for a specific ethnic or cultural group.[8] Indeed, many acculturation scales have been tailored towards the Hispanic/Latino community within the US.[13–16] Experiences within these groups may not be representative of other countries and ethnicities. In Canada and the United States, a large percentage of recent immigrants are from East and South East Asia, and South Asia (over 50% of all new immigrants to Canada).[17–19] One Canadian measure, the Vancouver Index of Acculturation, was developed specifically for use among Chinese-American/Canadian youth.[20] However, this Index was developed to evaluate culturally specific outcomes, such as *taijin kyofusho*, a Japanese construct similar to social anxiety and how individuals from collectivist societies adapt to individualistic societies. Thus, the applicability of this Index is unknown in broader populations.[20] To our knowledge, no other surveys exist for use among diverse samples of Canadian youth. Questionnaires need to be able to measure acculturation in individuals with diverse heritage culture norms and values in order to be practical, understandable, and informative. Once developed, these questionnaires can be used in large, population-based surveys to assess the impact of acculturation on health behaviours and outcomes.

The objective of this study was to develop and validate a short, self-report questionnaire that could be used to assess acculturation amongst ethnically diverse, bicultural, youth. Canada is a proponent of multiculturalism and a fitting research setting since multiculturalism supports the retention of an individual’s heritage culture, while encouraging integration into the dominant culture.[21] Canada also has a large proportion of young immigrants: almost 20% of all new immigrants to Canada are below the age of 14 and come from diverse regions of the world.[17,22] Thus, a questionnaire developed in Canada could have wide applicability to a broad range of countries and contexts.

## Methods and Results

### Study Overview

This study consists of three parts. Part A was a systematic literature review conducted in order to identify self-report questionnaires that have been used to measure acculturation among youth. Based on the review, a master list of candidate items that measure acculturation in young people was produced. In Part B, this list was reduced using a modified Delphi process to obtain consensus. Part B thus resulted in a brief acculturation questionnaire. Part C involved pilot testing of the newly developed questionnaire on a sample of 18–25 year old Canadians. Based on the results of pilot testing, the questionnaire was further refined by eliminating redundant items. Convergent validity and reliability estimates are reported for the final questionnaire. The methods and results from each part are presented consecutively below.

Parts A (literature review) and B (Delphi Process to Select Questionnaire Items) did not require formal ethics approval as per Queen’s University Health Sciences and Affiliated Teaching Hospitals Research Ethics Board policies. The pilot testing phase of the study (Part C) received ethics approval from the Queen’s University Health Sciences and Affiliated Teaching Hospitals Research Ethics Board (File # 6010933). Participants provided written consent using the online questionnaire system.

### Panel Selection

Two panels were assembled to provide input during the questionnaire development process. The expert panel (n = 5) reviewed the relevance of all studies identified by Part A, and ensured that the questionnaire developed in Part B had content validity i.e., that important domains of acculturation were included. Members of this panel included researchers with expertise in acculturation.

A student panel was also selected (n = 6). These students were selected to comment on whether the identified domains in Part B accurately reflected their acculturation experiences. Panelists were identified through cultural and religious clubs and societies at Queen’s University, Canada, and were sanctioned by the Queen’s University Alma Mater Society (student government). Inclusion criteria for the student panel were: 1) students could not be international students, and 2) had to be pursuing undergraduate education. It was believed that these students would be highly engaged with both their own and the Queen’s University community and thus be able to comment candidly and appropriately on acculturation.

### Part A: Literature Review

#### Methods

The review identified existing studies that included measurement of acculturation using self-report questionnaires administered to youth. Questionnaires were identified by searching academic databases (MEDLINE, EMBASE, PsycINFO, Social Science Citation Index and the Education Resource Information Center). Studies that used the word “acculturation” as a keyword or search term were linked to those that used the search terms “self-report,” “questionnaire,” or “survey” using an “AND” operator. Results were limited to English-language studies, published from 1990–2013, and of youth (< 18 years of age). To identify unpublished questionnaires in the grey literature, the websites of the World Health Organization, the Public Health Agency of Canada, Health Canada, EuroStat and the US Centers for Disease Control and Prevention were searched.

After removal of duplicates, titles and abstracts were screened. Studies were included if they: 1) measured acculturation using a self-report questionnaire, and 2) were of youth < 18 years of age. Studies were excluded if they: 1) measured acculturation in adults, 2) used a proxy measure of acculturation such as immigrant generation, time since immigration, or compared ethnic/cultural groups, 3) used qualitative methods, or 4) were unrelated to the measurement of acculturation, such as studies that validated an existing measure in a new population. If the population under study and/or the measure of acculturation used was unclear from the abstract, the study was (conservatively) included for full text review using the same criteria as above.

Questionnaires used for measuring acculturation in studies that met these criteria were identified and obtained. If the questionnaire was not available publicly, the corresponding author was sent an email to try to obtain a copy. Items from these questionnaires were extracted to form a master list of acculturation items. Duplicative items were collapsed into a single item and those inappropriate for a youth audience (e.g., items about marriage) were removed. These questions were then grouped into dominant/heritage culture pairs, and into domains using previous questionnaires and theory as a guide. The review was conducted by AK with input on the search strategy given by a Queen’s University librarian, the expert panel, and co-authors.

#### Results

**Fig 1:**
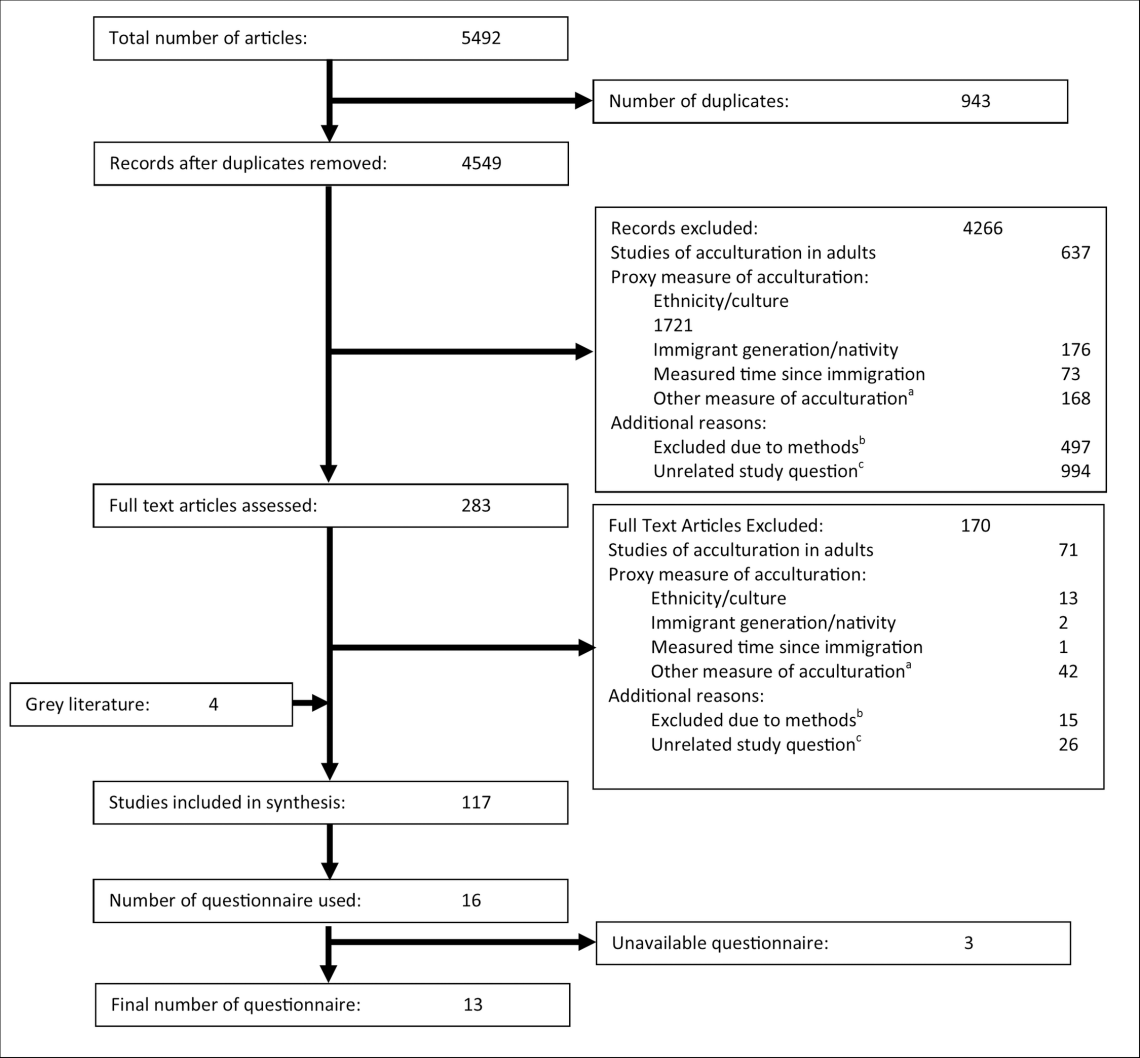
Flowchart for literature review. ^a^ These studies used a combination of measures to assess a proxy of acculturation, such as language of survey administration, or some combination of ethnicity, language and/or nativity. ^b^ These studies were excluded based on methodological grounds. They were either qualitative, mixed-methods, interview administered questionnaires or commentaries in journals. ^c^ These studies were unrelated to the current study. These included validating a questionnaire in a new population or did not investigate acculturation

The search identified 5492 studies, of which 943 were duplicates, leaving 4549 unique studies. Of these, 4266 were excluded based on a review of the title and abstract, leaving 283 studies for full text review (Fig 1). Full text review excluded 170 studies; 99 of which used either proxy measures of acculturation, were out of scope of the current study due to their methods, or because they answered an unrelated study question. The search of the grey literature revealed an additional 4 studies. A total of 117 articles met the inclusion criteria (Fig 1). These used 39 different questionnaires to measure acculturation, while 7 studies created their own questions to measure acculturation. Only 16 questionnaires were used in more than one study, and 13 were available to researchers (Fig 1).[15,20,21,23–32]

**Fig 2:**
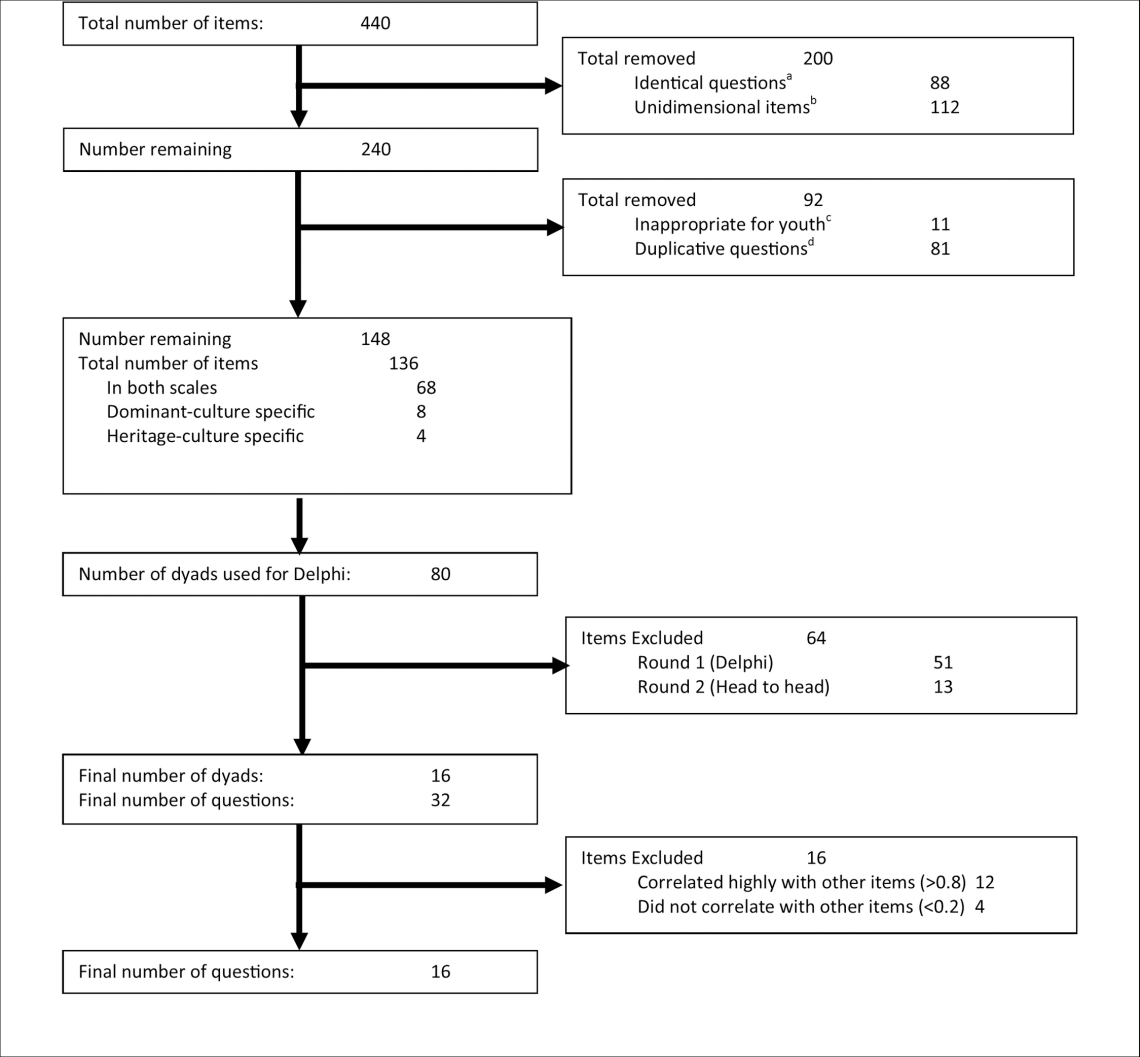
Flowchart for selecting items for inclusion in the questionnaire. ^a^ These questions were identical between questionnaires, i.e. “How well do you speak English”. ^b^ Theses questions were unidimensional, i.e. “Do you prefer to speak English or Spanish”. ^c^ These questions asked about items that would be beyond the scope of youth aged 10–16, such as “I communicate with my spouse in English”. ^d^ These questions were duplicative, such as “I speak English 1) at home, 2) at work, 3) with my mother, 4) with my father” etc.

Items from each of these questionnaires were extracted, yielding 440 items. After removal of identical (n = 88) and uni-dimensional items, i.e., those with response scales ranging from dominant culture to heritage culture, (n = 112), 240 questions remained (Fig 2). Questions deemed inappropriate for youth (n = 11) and deemed duplicative, i.e., referring to the same underlying construct (n = 81), were also removed. If necessary the wording of items was changed to ensure consistency of style within this questionnaire. The items on the Canadian, i.e., dominant culture, scale, were all changed to identify affinity for Canadian culture, while the items on the heritage scale were changed to say “my heritage culture.” This was necessary as some of the scales were developed for specific groups, such as Hispanic-Americans.[24,25,31] Finally, question response options were modified, if necessary, so that they all used the same 5 point Likert scale ranging from “strongly disagree” to “strongly agree.” Following this process, 148 items remained; 136 items in both scales, as well as 8 dominant-culture specific and 4 heritage-culture specific items (Fig 2). The questions were grouped into dominant/heritage culture dyads, which resulted in 80 total items, i.e., 68 with dominant and heritage culture analogues, 8 specific to the dominant culture, and 4 specific to the heritage culture.

These 80 items were grouped into four domains: 1) Personal, 2) Family, 3) Language, and 4) Social. The personal domain explores how much the respondent identifies with both Canadian, as well as their heritage cultures. The family domain explores the family’s level of acculturation. Language proficiency explores how comfortable the individual is speaking English as well as their heritage language. Lastly, the social domain asks individuals about those with whom they spend their free time, and how comfortable they are with individuals of the dominant culture and their heritage culture. The suitability of these domains was presented to the expert panel and universally approved. The end result of Part A was thus 80 questions covering 4 domains.

### Part B: Delphi Process to Select Questionnaire Items

#### Methods

The Delphi method was then used to refine and reduce the list of 80 potential questionnaire items developed in Part A.[33] The Delphi technique is a way of systematically eliciting and refining group judgements to build consensus and is particularly useful when consulting with experts and stakeholders with varied opinions and perspectives.[33,34] The 80 items were sorted by domain and uploaded to an online questionnaire system (FluidSurveys™, Ottawa, ON). All 11 panellists were asked to score each question on a nine-point scale from 1 (highly inappropriate) to 9 (extremely appropriate). Items were included if: 1) the median response was between 7–9, i.e., the top three response options, and 2) after discarding the highest and lowest responses for each item, the remaining nine responses were within a three point range.[35]

Remaining items then were subject to a pairwise comparison process. Each item was compared to all other items within the same domain and for each comparison panel members chose which item is more important to the study of acculturation among youth. The panelists’ responses were combined to form a master list, where items at the top were ranked the most important. *A priori*, it was decided that the top five items from each of the four domains would be retained for Part C in order to create a 40 item questionnaire.

#### Results

Of the 80 items presented to the Delphi panelists, 29 met both criteria. Sixteen items were in the personal domain, 8 were in the language domain, 3 were in the family domain, and 2 were in the social domain. The 5 items in the family and social domains were included for consideration in Part C as they were the only items in those domains that met the inclusion criteria. For the remaining 24 items, there were 148 head-to-head comparisons (120 in the personal domain; 28 in the language domain). From this, the top 5 rank ordered items were retained. Because there was a tie for the 5^th^ and 6^th^ top items in the language domain, 6 items were retained. Thus, 32 questions were retained for consideration in Part C (Fig 2).

### Part C: Item reduction, pilot testing and calculation of psychometrics

#### Methods

The 32 questions identified in Part B were pilot tested on a sample of young adults from Canada. The purposes of Part C were twofold. First, if items had high (> 0.8) correlations with other items, only one was retained, while those that had low (<0.2) correlations with other items were removed.[36–38] Using the remaining items the psychometric properties, i.e., convergent validity and test-retest reliability, as well as the underlying factor structure, were determined.

**Description of the sample:** The sample was recruited using “Survey Monkey Contribute”; Survey Monkey’s proprietary database of respondents.[39,40] Our sample was drawn from their Canadian partner network.[41] For this study, inclusion criteria were that respondents had to be: 1) residents of Canada; 2) identified as non-Caucasian, and 3) aged 18–25 years. This age group was a convenience sample, chosen to determine if the questionnaire demonstrated validity and reliability in a young adult population, before proposing pilot testing among school-aged youth. Respondents, in exchange for their time, could either 1) donate $0.50 to a charity of their choice, or 2) receive one entry for a weekly draw for a $100 gift card.[39] Respondents were categorized as White, Aboriginal, South Asian, Arab/West Asian, East/South East Asian, Black, Latin American and “other” using categories selected based on the 2006 Canadian Census of Population.[42]

**Item reduction:** Polychoric correlations were used to eliminate items that had very high correlations (r > 0.8) or very low correlations (r < 0.2).[43] Those with high correlations may be redundant, while those with very low correlations may not load on to the same factors as the other terms. Polychoric correlations are used when it is theorized that the underlying distribution of a variable is normally distributed, however, the variable itself is ordinal.[43] Response categories for the questionnaire exist on a 5 point Likert scale ranging from “strongly disagree” to “strongly agree” and thus this approach was appropriate.

**Factor analysis:** After elimination of items, exploratory factor analyses were conducted to determine the number of factors within which the items clustered. Factor analysis was conducted in SAS 9.4 using PROC FACTOR, the maximum likelihood method and varimax rotation. Parallel analysis methods were used to confirm the factor structure using SAS syntax created by O’Connor.[44] For each domain, internal consistency was evaluated using Cronbach’s alpha.

**Convergent validity:** To test the convergent validity of the survey, the mean score for each domain was calculated by immigrant generation and age at immigration.[45] Immigrant generation was defined as: 1) born abroad (1^st^ generation), 2) born in Canada with at least one parent born abroad (2^nd^ generation), and 3) born in Canada with both parents born in Canada (3^rd^ generation). Age at immigration was dichotomized to those who immigrated before age 12, and those who immigrated at the age of 12 or older. This was chosen as it is the age at which the ability to learn new languages starts to decline.[15] It was hypothesized that dominant culture score will be positively associated with increasing immigrant generation, while heritage culture score will be negatively associated with increasing immigrant generation and higher among those who immigrated after age 12.

**Test-retest reliability:** To assess test-retest reliability of the questionnaire items, participants were invited to complete the survey again after two weeks. In addition to comparing the mean baseline and follow-up scores, intra-class correlations were calculated. Finally, Bland Altman plots were used to confirm that the findings were reliable over the range of response values.[46–48]

All statistical analyses were conducted in SAS 9.4 (SAS Institute, Cary, NC), while Bland Altman plots were created in Excel 2013 (Microsoft Corporation, Redmond, WA).

#### Results

**Sample Characteristics:** Two hundred and eighty two people responded to the survey. Of these, 248 answered all 32 questions (87.9%). Of these 248 respondents, a further 101 completed the survey again two weeks later. The baseline sample (n = 248) included more females than males (54.8%), and reported a mean age of 20.9 years (SD: 2.5) (Table 1). They identified mainly as East and South East Asian (27.8%), South Asian (17.7%) and Black (13.7%). The majority were 1^st^ (33.5%) or 2^nd^ generation (52.0%) immigrants. Of those who were born abroad, the mean age at immigration was 10.8 years (SD: 6.0). Approximately half spoke a language that they identified as their heritage language (53.6%) and answered the questions on heritage language ability.

**Table 1:** Description of the sample used for pilot testing.

	Baseline	Follow up
**N**	248	101
**Categorical Measures**	**N (%)**	**N (%)**
**Gender**		
Male	112 (45.2)	46 (45.5)
Female	136 (54.8)	55 (54.5)
**Ethnicity**		
White	15 (6.0)	5 (5.0)
Aboriginal	10 (4.0)	5 (5.0)
South Asian	44 (17.7)	17 (16.8)
Arab/West Asian	13 (5.2)	4 (4.0)
East/SE Asian	69 (27.8)	39 (38.6)
Black	34 (13.7)	11 (10.9)
Latin American	7 (2.8)	0 (0.0)
Other	56 (22.6)	20 (19.8)
**Immigrant Generation**		
1st generation	83 (33.5)	27 (26.7)
2nd generation	129 (52.0)	57 (56.4)
3rd generation	36 (14.5)	17 (16.8)
**Speak a language in addition to English**		
Yes	130 (52.4)	56 (55.4)
No	118 (47.6)	45 (44.6)
**Age**	**Mean (SD)**	**Mean (SD)**
Current age	20.9 (2.5)	21.2 (2.4)
Age at immigration	10.8 (6.0)	10.0 (5.7)

The baseline sample was similar to those who responded at follow-up, with no gender differences (54.8% vs 54.5% female), or age (20.9 (sd: 2.5) vs (21.2 (sd: 2.4) years old) (Table 1). The baseline sample included fewer people who identified as East and South East Asian (27.8%) compared to follow-up (38.6%), and more 2^nd^ generation respondents (33.5% vs 26.7%).

**Item reduction:** Of the 32 items that were tested, 12 correlated highly (r>.80) with other items, while 4 did not (r < .20). These items were eliminated so that 16 items remained in the Bicultural Youth Acculturation Questionnaire (see S1 Questionnaire).

**Factor analysis:** The results of the factor analysis performed on the 16 items are shown in Table 2. Two separate exploratory factor analyses were conducted. The first factor analysis employed the full sample (n = 248) and used all of the dominant and heritage culture questions but not the language domain questions. The second factor analysis was limited to the 130 participants who spoke their heritage language. This factor analysis used the dominant and heritage culture questions and the language domain questions (n = 130).

**Table 2:** Results of factor analyses, psychometric properties of subscales.

	Factor Loading^a^	Test Statistics
**Dominant culture Domain**		
In general, I feel comfortable speaking English	.74	Eigenvalue^b^: 12.10/ 8.15
I believe in Canadian values	.74	Cronbach’s α: 0.85
I have a lot of pride in Canadian culture and its accomplishments	.76	
I was raised in a way that was consistent with Canadian culture	.53	
When I was growing up, I was exposed to Canadian culture.	.57	
I am interested in having Canadian friends outside of my heritage culture	.79	
**Heritage Culture Domain**		
I believe in the values of my heritage culture	.64	Eigenvalue^b^: 6.10/ 4.79
I have a lot of pride in my heritage culture and its accomplishments	.73	Cronbach’s α: 0.83
I was raised in a way that was consistent with my heritage culture	.69	
When I was growing up, I was exposed to my heritage culture	.72	
I am interested in having friends from my heritage culture.	.58	
**Heritage Language Domain**		
In general, I feel comfortable speaking my heritage language	.58	Eigenvalue^b^: 3.00 /
With my friends, I feel comfortable speaking my heritage language	.77	Cronbach’s α: 0.86
I enjoy watching TV programs in my heritage language	.62	
I enjoy reading books in my heritage language	.68	
My thinking is done in my heritage language	.84	

^a^ Factor loadings are based on the 3 factor solution (n = 130)

^b^ Eigenvalues are presented for the: 3 factor solution (n = 130) / 2 factor solution (n = 248)

For the first factor analysis, which was based on the full sample, the Scree test and results from the parallel analysis indicated a two factor solution. The two factors were named: 1) dominant culture, and 2) heritage culture. All items within each factor reported rotated factor loadings of > 0.5 and Cronbach’s alpha values of > 0.8. For the second factor analysis, which was based on the 130 participants who spoke the heritage language, a three factor solution emerged (Table 1). These three factors were named: 1) dominant culture, 2) heritage culture, and 3) heritage language. The factor pattern for the dominant culture and heritage culture domains was identical when comparing the results from the first factor analysis and the second factor analysis, i.e., speaking their heritage language did not impact the dominant and heritage culture domains. The magnitude of the factor loadings was very similar between factor analyses. For example, the factor loading for “I believe in Canadian values” was 0.74 in the three factor solution, compared to 0.81 in the two factor solution. The third factor (heritage language), emerged as a distinct factor in the three factor solution. For each of the three factors, all constituent items had factor loadings that exceeded 0.5, and all three factors had high internal consistency with Cronbach’s alpha values of > 0.8 (Table 2). These three factors therefore replaced the four domains used in Part B.

**Convergent validity:** Dominant domain score increased with increased generational status, with third generation youth reporting the highest mean score (third generation: 4.40 (95% CI: 4.19–4.61) vs first generation: 3.69 (3.49–3.89)). Mean heritage language score was lowest among second generation immigrants compared to first and third generation peers (3.05 (2.84–3.26) vs. 3.43 (3.17–3.69) and (3.94 (3.17–4.71) respectively). Mean heritage domain score was high among all three immigrant generation groups (Table 3).

**Table 3:** Means, confidence intervals and group differences of subscales by immigrant generation and age at immigration.

		Dominant domain		Heritage domain		Heritage language
	n	Mean (95% CI)	n	Mean (95% CI)	n	Mean (95% CI)
**Immigrant generation**						
1st Generation	83	3.69 (3.49–3.89)	83	4.02 (3.85–4.20)	58	3.43 (3.17–3.69)
2nd Generation	129	4.13 (4.00–4.26)	128	3.97 (3.84–4.10)	73	3.05 (2.84–3.26)
3rd Generation	36	4.40 (4.19–4.61)	36	3.94 (3.71–4.18)	7	3.94 (3.17–4.71)
p-value^a^		< .0001		.85		.0011
**Age at Immigration**						
< 12 years old	50	3.64 (3.34–3.95)	50	3.89 (3.64–4.14)	33	3.03 (2.70–3.35)
> 12 years old	33	3.77 (3.55–3.98)	33	4.23 (4.01–4.46)	25	3.97 (3.64–4.30)
p-value^b^		.5		.056		.0001

^a^ ANOVA of overall group differences

^b^ t-tests of differences between the two age groups

Table 3 shows the mean score stratified by age at immigration. Youth who immigrated after the age of 12 reported similar scores for the dominant or heritage domain as peers who immigrated before the age of 12. However, youth who immigrated after the age of 12 reported significantly higher mean scores on the heritage language domain compared to those who immigrated before the age of 12 (3.97 (3.64–4.30) vs 3.03 (2.70–3.35) respectively) (Table 3).

**Fig 3:**
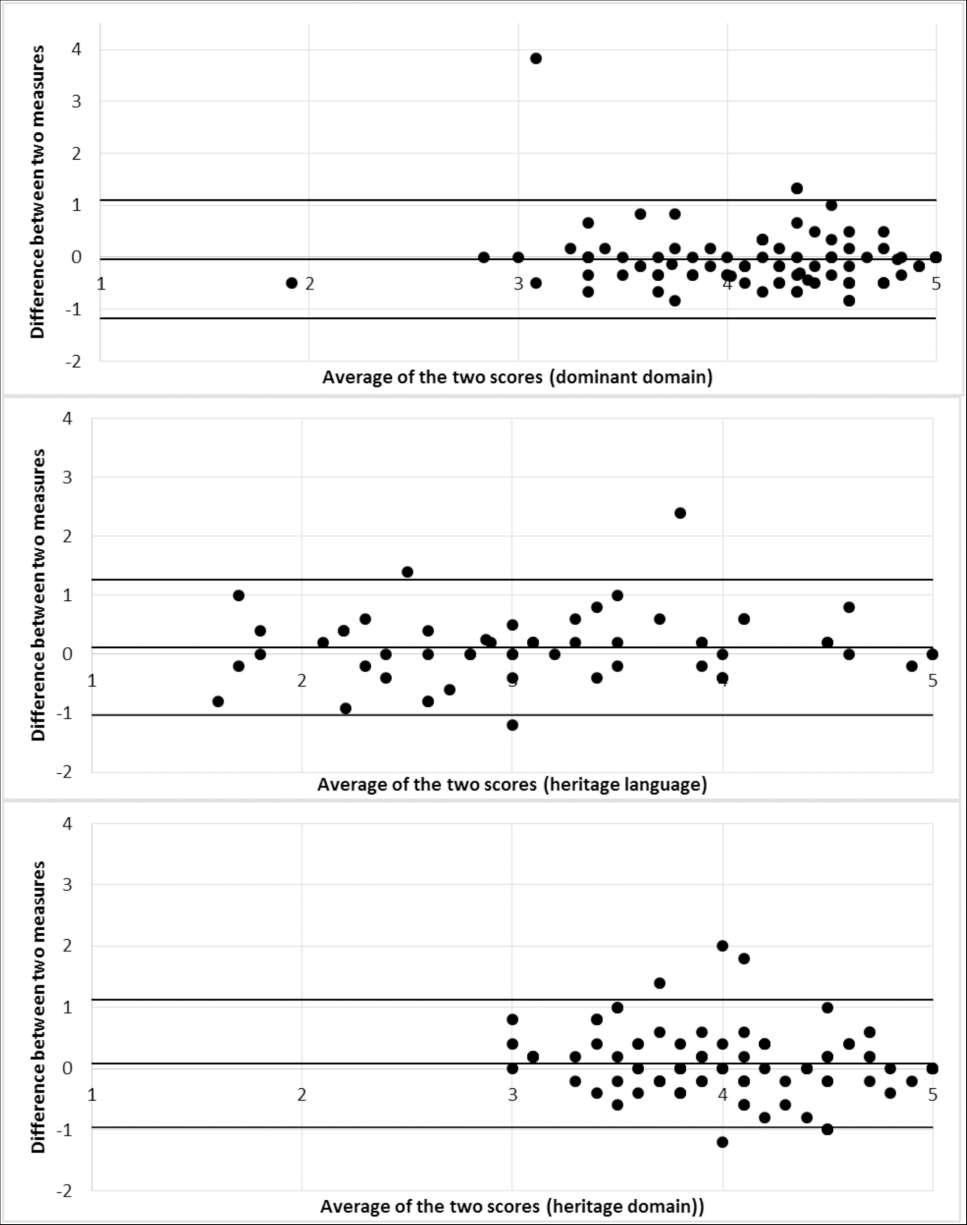
Bland-Altman Plot of differences between baseline and follow up over the mean response value for each domain.

**Test-retest reliability:** Two measures of test-retest reliability were calculated for the participants who completed the survey again after two weeks (n = 101). All three domains reported acceptable reliability with intra-class correlations above .60 (Table 4). Finally, Bland-Altman plots were created for each domain. The 95% limits of agreement were approximately ± 1 on a 5 point scale (Fig 3).

**Table 4:** Test-retest reliability by domain.

	Dominant Domain	Heritage Domain	Heritage Language
n	97	96	64
Intra-Class Correlation	.62	.66	.81

## Discussion

In this study we describe the development and validation of the Bicultural Youth Acculturation Questionnaire. The final questionnaire that was developed had good psychometric properties, as well as convergent validity with immigrant generation and age at immigration. An important and unique finding of this study was that English language ability could not be used to identify high and low acculturated youth due to ceiling effects.

This study determined the convergent validity of this questionnaire using two common proxy indicators of acculturation: immigrant generation and age at immigration. Our study found convergent validity with these indicators, with some caveats. While mean dominant domain score increased with higher immigrant generation, showing stronger affinity for the Canadian dominant culture, mean heritage domain score did not change with increasing immigrant generation. Integration into the dominant culture and retention of heritage cultural norms is the hope of multiculturalism.[21] For first generation immigrants, dominant domain scores were comparable between those who immigrated before and after the age of 12, while both heritage domains reported higher mean scores for those participants who immigrated at the age of 12 years or older, indicating a higher affinity for the heritage culture. This has particular relevance for those studying health outcomes among youth. Depending on the proposed mechanism underlying the health behaviour, i.e., whether the health outcome is a result of heritage cultural norms or the result of the adoption of dominant culture norms, the use of either immigrant generation or age at immigration may be appropriate. We found that generational status can be used as a proxy for dominant domain adoption, while age at immigration can be used as a proxy for heritage domain retention. A rationale would need to be provided for the appropriateness of these measures, if they are being used as a proxy for acculturation, in future studies.

We had two unique findings in the use of language as a measure of acculturation. The first was that using English language proficiency as a proxy measure of acculturation is not particularly effective among youth, even though it has been shown to have utility among adults.[49] In our sample, almost all respondents reported being comfortable with the English language, and thus this could not be used as a way of separating youth into high vs low acculturated groups. This is not surprising as over half of our sample immigrated to Canada when they were quite young (< 12 years of age) and would have completed English as part of their high school education. A response bias may also be driving this observation, with those having higher English language ability responding to the survey. There was a second important finding regarding heritage language. Of those youth who reported having a heritage language, second generation youth reported the lowest mean language score. Two potential explanations exist for this finding. The first possibility for this is due to the small number of youth in the third generation category (7 reported having a heritage language). A second related explanation is residual confounding as a result of self-selection by respondents to answer these questions. Third generation youth who responded to these questions may have received formal training or instruction in their heritage language, and thus report a high level of competence. In contrast, third generation youth who did not receive such instruction may not have responded to these questions as these youth do not speak their heritage language. This would manifest as a higher mean heritage language score among third generation youth.

Future use of a more precise assessment of acculturation could allow researchers and public health professionals to further disentangle the mechanisms behind health changes that occur with acculturation, and why discrepancies exist between groups under study. Currently, immigrant generation and age at immigration are used, and provide a crude, but useful, indicator of acculturation with clear public health relevance.[9] Measurement of health behaviours among new immigrants or by race and ethnic groups allows for high priority populations to be identified. Our research supports the use of these proxy measures, but notes that there is considerable heterogeneity within these groups, supporting previous studies.[11,12] Use of a more precise measure will help tease out whether there is a cultural explanation for health differences, or whether this is because of an issue associated with immigration, such as knowledge of health services.[50] Our questionnaire provides a short and practical method of collecting this information.

This study has several strengths. The rigorous methodology applied has created a questionnaire with high content and convergent validities for the measure of youth acculturation, with input from both experts and young people. The domains showed convergent validity with immigrant generation and age at immigration. Finally, the pilot testing process used a sample of youth from across Canada of different ethnicities, linguistic abilities and immigrant generations, showing it has broad applicability.

The study also has some limitations. In trying to create a short measure of acculturation, we may have excluded some domains. For example, dietary acculturation was not included in the list of final questions. The results for test-retest reliability suggest that while the instrument has high ICC, this may be the result of the lack of variation in the dominant and heritage domains. The Bland-Altman plot shows that the results almost all exist in the 3–5 point range, with only the heritage language domain utilizing the full range of response options. This is of concern, as the 95% limits of agreement are ±1, encompassing half of the responses. The lack of variation may have been the result of response bias of those choosing to be included in our sample. For someone to participate, they needed to have access to the internet, be signed up for Survey Monkey Audience, respond in English, and have time to respond. These individuals may be more integrated into Canadian society and their dominant culture, and thus more likely to respond to a questionnaire on acculturation. This would result in high scores on each domain, and so the scores may not be generalizable to the immigrant population.

A related limitation was the sample used for pilot testing. Youth in our sample were aged 18–25 years, and were a convenience sample recruited through the commercial service “Survey Monkey.”[39,40] This sample was admittedly older than the target age group (10–16 years of age) and their responses may reflect different priorities surrounding acculturation than this younger target age. Our sampling goal was to identify a general population of young people in Canada who experienced a diverse level of acculturation. Therefore, we excluded sampling from religious institutions or ethnic-based community groups, as these youth would be likely to score highly on their heritage domain scores. In order to sample from the community, we considered recruiting our sample from middle and high schools in Ontario (Grade 6 through 10). This would provide the most representative sample of the population, with a range of acculturative experiences. However, this approach was not feasible given available study resources, including time and ability to recruit school boards, schools, and students from a range of educational backgrounds. Assuming that 39.4% of Canadians are 1st and 2nd generation immigrants, and approximately 77% of youth would respond to the survey, (250 / .394 / .77 =) 824 students would need to be approached to obtain a final sample of 250 new immigrants.[51–53] This was not feasible. The Survey Monkey sample proved to be diverse and represent a heterogeneous range of acculturation experiences. We argue that the questions asked, i.e., “how they were raised”, “whether they want friends from their heritage culture”, and their “belief in Canadian values”, are applicable to both older and younger youth (a direct consequence of the instructions given to the Delphi panellists). Further validation work among the younger population is required to confirm the resultant factor structure. Finally, the survey was only available in English. Consequences of this were twofold. In addition to the reasons stated before, there may be a lack of variability in the dominant domain questions as these individuals are already comfortable speaking English. Furthermore, since the official languages of Canada are English and French, the survey needs to be translated into French in order to be used across Canada.

## Conclusion

We have developed and pilot tested a short and effective questionnaire that measures acculturation among Canadian young people. It reported convergent validity with other indicators of acculturation, and the scales have reported high reliability. If included in general health surveys of youth, our questionnaire can help capture the process of acculturation more completely, and improve the understanding of the mechanisms underlying the health of Canadian ethnic minorities and immigrants.

## Supporting Information

S1 Questionnaire The Bicultural Youth Acculturation Questionnaire. (DOCX)
