# Supplementary material for: Development and Validation of the Bicultural Youth Acculturation Questionnaire
Source: PLoS One. 2016 Aug 24;11(8):e0161048. doi: 10.1371/journal.pone.0161048 (PMC4996452; doi:10.1371/journal.pone.0161048)
Supplement: S1 Questionnaire — (DOCX) [file pone.0161048.s001.docx]

**The Bicultural Youth Acculturation Questionnaire**

The purpose of this questionnaire is to measure acculturation in Canada. Acculturation is the process of change due to contact between people from different cultures. First, you will be asked a series of question about how you relate to the dominant culture (Canada). You will then be asked about your heritage culture.

**Dominant culture Questions**

The “dominant culture” refers to Canadian values, norms and beliefs. For each question, please select one of the six response options.

|  |  | Strongly disagree | Disagree | Neutral | Agree | Strongly Agree | Prefer not to answer | Not applicable |
| --- | --- | --- | --- | --- | --- | --- | --- | --- |
| 1 | In general, I feel comfortable speaking English | o | o | o | o | o | o | o |
| 2 | I believe in Canadian values | o | o | o | o | o | o | o |
| 3 | I have a lot of pride in Canadian culture and its accomplishments | o | o | o | o | o | o | o |
| 4 | I was raised in a way that was consistent with Canadian culture | o | o | o | o | o | o | o |
| 5 | When I was growing up, I was exposed to Canadian culture. | o | o | o | o | o | o | o |
| 6 | I am interested in having Canadian friends outside of my heritage culture | o | o | o | o | o | o | o |

**Heritage Culture Questions**

The “heritage culture” refers to your country of origin, or the ethnic group you identify with. Examples include Chinese, Indian, Filipino or another culture.

7. Do you speak a language that you consider to be the primary language of your heritage culture? If you speak multiple languages, please pick the one you speak most often with family and friends.

If yes, please click here ⬜ (Go to Question 8)

If no, please click here ⬜ (Go to Question 14)

8. Do you speak a language that you consider to be the primary language of your heritage culture? If you speak multiple languages, please pick the one you speak most often with family and friends.

|  | Arabic |  | Japanese |
| --- | --- | --- | --- |
|  | Armenian |  | Korean |
|  | Chinese |  | Persian |
|  | English |  | Polish |
|  | French |  | Portuguese |
|  | French Creole |  | Russian |
|  | German |  | Spanish |
|  | Greek |  | Tagalog |
|  | Gujarati |  | Urdu |
|  | Hindi |  | Vietnamese |
|  | Italian |  | Other (please specify) |

For each question, please select one of the six response options.

|  |  | Strongly disagree | Disagree | Neutral | Agree | Strongly Agree | Prefer not to answer | Not applicable |
| --- | --- | --- | --- | --- | --- | --- | --- | --- |
| 9 | In general, I feel comfortable speaking my heritage language | o | o | o | o | o | o | o |
| 10 | With my friends, I feel comfortable speaking my heritage language | o | o | o | o | o | o | o |
| 11 | I enjoy watching TV programs in my heritage language | o | o | o | o | o | o | o |
| 12 | I enjoy reading books in my heritage language | o | o | o | o | o | o | o |
| 13 | My thinking is done in my heritage language | o | o | o | o | o | o | o |

For each question, please select one of the six response options.

|  |  | Strongly disagree | Disagree | Neutral | Agree | Strongly Agree | Prefer not to answer | Not applicable |
| --- | --- | --- | --- | --- | --- | --- | --- | --- |
| 14 | I believe in the values of my heritage culture | o | o | o | o | o | o | o |
| 15 | I have a lot of pride in my heritage culture and its accomplishments | o | o | o | o | o | o | o |
| 16 | I was raised in a way that was consistent with my heritage culture | o | o | o | o | o | o | o |
| 17 | When I was growing up, I was exposed to my heritage culture | o | o | o | o | o | o | o |
| 18 | I am interested in having friends from my heritage culture. | o | o | o | o | o | o | o |
